# Attention, memory and consciousness: Historical context, evolution, and impact of Jacoby’s process dissociation procedure

**DOI:** 10.3758/s13421-025-01835-5

**Published:** 2026-01-30

**Authors:** David Balota

**Affiliations:** Department of Psychological and Brain Sciences, Washington University, St. Louis, MO 63130 USA

**Keywords:** Attention, Consciousness, Familiarity in recognition memory, Memory, Automaticity, Automatic processing

## Abstract

This article focuses on the last 25 years of the 20^th^ century when Larry Jacoby had an extraordinary influence in the areas of attention and memory. During a short 3-year period between 1977 and 1980, four benchmark papers in cognitive psychology documented *qualitative* distinctions between automatic and attention demanding processes. These studies reflected the zeitgeist for Jacoby’s early work extending this distinction to memory. Jacoby developed ingenious experimental paradigms to distinguish automatic and attentional processes and boldly explored unconscious influences, when most experimental psychologists were avoiding using such an introspective term. Although this work was powerful, it did not afford a way of quantifying conscious and unconscious contributions to performance, which ultimately led to Jacoby’s process dissociation procedure (PDP). Strong assumptions (such as independence of the two processes) were necessary and these have generated controversy in the field. Although there are limitations, the PDP has been remarkably generative in not only understanding the relation between attention and memory but also has been widely extended to other domains within psychology. The present article attempts to capture the energy and enthusiasm in the field during this period in history, which continues to serve as foundational for work in cognitive science and neuroscience.

A major goal of experimental psychology is to move beyond our intuitions and commonsense explanations of behavior. If the discipline cannot move beyond such explanations, then it would lose its currency. There are many phenomena in psychology that have *folk psychological* explanations after some simple intuitive analyses.

Jacoby’s work is at the forefront of moving beyond folk psychology. His creative experimental designs afforded deep insights into cognition and have played a critical role in developing unifying theories of memory, attention, and consciousness, and have naturally been extended to social psychological processes, aging, and cognitive neuroscience. Although Jacoby’s work has been influential in multiple domains of cognition, this article will focus on dual-process theory, which decouples more folk psychological conscious attentional processes from more hidden automatic unconscious processes. In pursuit of this goal, there are four parts to this paper. First, I will provide a review of the zeitgeist that set the stage for Jacoby’s dual process emphasis. Second, I will describe Jacoby’s specific empirical work that served as a background for the development of his Process Dissociation Procedure (PDP). Third, I will discuss the PDP and how it has been used across different domains. Fourth, I will briefly discuss the influence of PDP, via extensions by one of his students and close collaborator, Andrew Yonelinas. Intertwined across these sections will be discussion of major assumptions and critiques of the approach. As one will see, the focus is on work conducted between 1975 to 2000, by both Jacoby and other researchers. The hope is to capture the context and evolution of Jacoby’s work and why this work generated remarkable enthusiasm during this period that continues today. This is not intended to be a comprehensive review, which would be far beyond the limitations of a single paper, but rather a paper that provides a historical context for a major component of Jacoby’s work (for more detailed discussions, see Yonelinas, 2002; Yonelinas & Jacoby, 2012).

## Zeitgeist

The insight that much of cognition involves both a habit-based process along with a more attention based reasoned process is clearly not new and indeed was recognized by James (1890). Moreover, a folk psychological analysis might easily identify these two modes, as reflected in everyday language by terms such as running on autopilot, gut response, knee-jerk, in which one behaves in a manner that would *not* always be predicted by conscious deliberation. Although dual process perspectives have been around for many years, and indeed a bit of folk psychology might appear consistent with two modes of behavior, one might question whether these are indeed *qualitatively* different modes of processing. For example, an alternative perspective might suggest that different cues come on at different points in time, and some cues are more salient that they appear to drive a behavior (i.e., a knee-jerk response). For example, consider a threatening stimulus (a pistol) that may come on relatively quickly because of its salience/relevance, while other cues (the face of a person holding the pistol) may come on more slowly. Such a model would not demand *qualitatively* distinct processing modes but rather may involve the same processing mode with different cues becoming available across time that have different levels of salience. However, in contrast to this more continuous perspective, there were benchmark papers across a short 3-year span during the 1970s that provided compelling experimental and theoretical work decoupling what appeared to be *qualitatively* distinct processing modes. Given the timing of these studies, and the clear relevance to his perspective, it is useful to briefly describe these projects to capture the zeitgeist during Jacoby’s early work.

Shiffrin and Schneider’s (1977, also see Schneider & Shiffrin, 1977) work on search processes was a powerful demonstration of qualitative differences between automatic (more habit like) processes and attention driven processes. In order to experimentally produce habit-like behavior, participants engaged in visual search tasks in which the items that served as targets and those that served as distractors were either consistent or varied across trials. In the varied search conditions, one finds the standard increasing response latencies as a function of the number of items in the search set. For example, it takes more time to search a display with four items in the display than two items in the display. This is clearly consistent with our folk intuitions (i.e., it should take more time when there are more alternatives to look at). Remarkably, however, there was a qualitatively different search pattern after hundreds of trials of consistent mapping—namely, the consistent mapping afforded search performance that was independent of the number of items in the search set. Clearly, our intuitions would not predict that participants will be able to search for a target equally when two items are in the search set compared with when four items are in the search set. Hence, these researchers provided empirical evidence of how one can develop qualitatively different processes within an experimental context by providing consistent mapping across trials. They argued that automatic processes do not demand attentional capacity and are triggered by appropriate inputs due to long-term memory representations that develop with consistent mapping. Hence, the target appears to pop out in the background of distractors, independent of the number of distractors. In contrast, controlled processes require attention and are capacity limited and so each additional item to be searched produces a cost in performance (i.e., response latency). This powerful demonstration and theoretical analyses solidified the distinction between these two qualitatively distinct modes of processing.

The second benchmark paper by Neely (1977) was published the very same year. Based on Posner and Snyder’s (1975) theory regarding automatic and controlled processing, Neely experimentally demonstrated a dissociation between automatic and controlled processing via retrieval from a semantic memory system. That is, instead of examining automatic processing as it develops within an experimental setting as in the Schneider and Shiffrin research, Neely used a semantic priming paradigm to examine this distinction in retrieval from a pre-existing semantic network. He was able to dissociate an automatic spreading activation process from a more volitional attention limited capacity process, via manipulations of both the time available to process the primes and the direction of attention via instructions to the participants regarding what they should consciously expect based on the prime identity. Very simply, the priming effects at short prime-target stimulus-onset asynchronies (SOAs) were driven by pre-existing prime–target semantic relationships independent of conscious expectancies (e.g., BIRD–robin was faster than Furniture–robin, independent of a condition in which participants were told to expect types of furniture when the prime word was BIRD), whereas the priming effects at longer SOAs were driven by where attention was directed due to the instructional manipulation independent of the pre-existing semantic relationship (e.g., BIRD–robin actually produced *inhibition* if participants were told to think of types of Furniture when Bird was presented). This remarkable dissociation provided a qualitative distinction between a fast acting automatic spreading activation mechanism and a slower acting attentional mechanism.

Two years later, Hasher and Zacks (1979) published an influential paper showing that the automatic and attentional distinction can also be extended to episodic memory performance and predictably varied as a function of individual differences. They reviewed and provided novel experimental evidence for the notion that automatic memory processing (e.g., frequency judgments regarding the number of times an earlier stimulus was presented) appears to be uninfluenced by instructional set and is relatively consistent across children, young adults, older adults, depressed individuals, and individuals who are experiencing stress. In contrast, attention demanding memory processes, such as free recall, are influenced by instructional set and varied across these participant groups. This combination of empirical work along with a comprehensive review of the literature was particularly influential in connecting the work on attention and automaticity to memory performance across different groups of participants, an approach that Jacoby used in his later work especially with older adults.

Jacoby repeatedly cites the above work in his important papers in the 1980s. In his comprehensive review of dual process perspectives, Yonelinas (2002, p. 448) argued that the automatic/controlled distinction is one of the unique characteristics of the Jacoby perspective. Other dual process models do not explicitly address the automatic/controlled distinction, although most are broadly consistent with this claim (i.e., one process is relatively more automatic and the second process being relatively more effortful). One might argue that models using the qualifiers “relatively more” lose the strength of the strong empirical dissociations in the papers described above. Jacoby embraced this distinction in much of his work.

At the same point in time, there was important work by Mandler and colleagues, which led to an important paper in 1980. Mandler (1980) also argued for separable processes being involved in recognition memory. Specifically, Mandler suggested that recognition memory could reflect the detection of familiarity (due to intraevent organization) along with a second context retrieval process (involving interitem processing). Mandler’s classic example of seeing one’s Butcher on a Bus naturally captured this distinction. Specifically, one might see their Butcher on a Bus and in some sense recognize the person due to a sense of familiarity regarding that person, but not be able to access the knowledge of where they recognize them from (which demands more explicit search and retrieval). Mandler provided procedures for mathematically decoupling the contributions of the two processes (see also Atkinson & Juola, 1974). Mandler’s work was important in leading to Jacoby’s (1991) influential PDP paper and is repeatedly cited in Jacoby’s own work.

These four highly influential papers were clearly not the only papers that provided dissociations, but they are particularly noteworthy because of their lasting impact (e.g., each of these papers has been cited at least 4,000 times according to Google Scholar) in providing evidence of strong qualitative distinctions between automatic and attention driven processes across a wide set of paradigms and participant groups. The context was set for new creative empirical and theoretical work extending our understanding of these two modes of processing to episodic memory performance. The zeitgeist was perfectly set for Jacoby’s creative empirical contributions.

## Jacoby’s early dual process empirical work

In extending the distinction between automatic vs. attention demanding processing to memory processing, Jacoby was also influenced by the classic work by Warrington and Weiskrantz (1968, 1973, 1978) with patients who had dense amnesia. Much of this work distinguished between performance on direct tasks that demand memory for a previous event (e.g., did you see this word earlier in the experiment) and indirect tasks that only demanded some perceptual operation (e.g., complete the fragment _ I _ R _ _ Y, with an acceptable word), without an accompanying direct memory decision. The important finding here is that amnesic individuals benefit from earlier being presented items that are answers for indirect tests, while failing the direct task of explicitly recognizing that the item was earlier presented in the experimental session. Similar dissociations have been found in many other tasks (e.g., Corkin, 1968). One approach to theorizing about such dissociations is to consider distinct levels of awareness demanded by the different tasks. Amnesic individuals only have a deficit in processes demanded by “aware” memory for the earlier presented item, whereas the indirect tasks might benefit from “unaware” perceptual tuning based on an item’s earlier presentation.

Jacoby and Dallas (1981) attempted to provide similar dissociations reported by Warrington and colleagues in studies of healthy young adults. The amnesic work and the work on automaticity clearly set the stage for one of Jacoby’s first major papers in this area. Jacoby and Dallas reported eight experiments and theoretical analyses, which showed that certain manipulations such as depth of processing (e.g., anagram solution vs. pronunciation during encoding) produced the expected strong effects on a direct test of episodic recognition, but little if any effect on an indirect test of identifying briefly presented masked words in a perceptual identification task. Importantly, however, Jacoby and Dallas also found some parallel effects of encoding manipulations on recognition memory and perceptual identification. Repetition of words, spacing of words, and the frequency of the words in the language (high frequency vs. low frequency words based on frequency corpora) all modulated both perceptual identification *and* recognition performance. Because of both the isolated effects of some manipulations on recognition memory, with no corresponding effect on perceptual identification (e.g., depth of processing manipulations), along with the parallel effects of other encoding manipulations on both recognition memory and perceptual identification, Jacoby and Dallas argued that recognition likely involves both an automatic relative familiarity-based process (reflected by the parallel effects across recognition and identification performance), along with a more attention-based retrieval of context (reflected by the isolated effects on recognition memory). They suggested that it is *relative* familiarity that is critical as opposed to absolute familiarity because, compared with high-frequency words, low-frequency words are more influenced by a study episode (which changes relative familiarity) in both recognition and perceptual identification. They also mention that self-reports appeared consistent with the influence of relative familiarity because participants sometimes reported that an old item “jumps out” (see Jacoby & Dallas, 1981, p. 308) from the background, similar to targets that have received hundreds of search trials in the Schneider and Shiffrin (1977) studies described above.

One might wonder why the Jacoby and Dallas paper was so influential (more than 4,000 citations) given that there were similar arguments regarding dual process perspectives already available. There are many reasons. First, as noted, this paper did not rely on results from studies with patient populations (e.g., amnesics) to argue for distinct processes, but developed creative paradigms to use with healthy young adults. Second, the task specific effects could be interpreted within the important transfer appropriate processing perspective  (Morris, Bransford & Franks, 1977), which Jacoby advocated in his earlier work. Third, because of the combination of both parallel and task specific effects of different manipulations, this work also strongly supported the notion that no task is *process pure*, which as we shall see is a guiding principle of his work. Fourth, and importantly, this paper clearly demonstrated Jacoby’s wide spanning scholarship, connecting to important ongoing work in other domains. This is a common theme in Jacoby’s work. For example, Jacoby and Dallas noted the importance of Nisbett and Wilson’s (1977) distinction between introspective based performance and more direct influences on performance. They suggested that recognition judgments can be viewed as an introspective report of the participant saying that a stimulus appears “old,” whereas, perceptual identification does not involve this introspective component (i.e., either the stimulus can or cannot be identified). Jacoby and Dallas also suggested that Tversky and Kahneman’s (1973) important work on heuristics nicely dovetailed with their results on automatic relative fluency. Moreover, they also pointed out that some seemingly perceptual tasks can also involve both automatic and attentional processes and specifically noted the lexical decision task in which participants make speeded word/nonword decisions on letter strings. Here they suggested that lexical decisions could be based on a familiarity process or a process accessing the meaning of the stimulus. This possibility predated a two-process model for lexical decision three years later proposed by Balota and Chumbley (1984), based on the Atkinson and Juola (1974) two process model.

Following the Jacoby and Dallas paper, Jacoby further developed and refined his conceptions of the two processes. An important contribution was his more explicit description of automatic and attentional influences on memory, and tying the former to *unconscious* influences. There are two major papers published in 1989. In Jacoby and Whitehouse (1989), the authors begin with an example from Titchener (1928), who described false recognition or paramnesia as an illusion of memory that is produced by “a disjunction of two distinct processes that are normally held together in a conscious present” (p. 126) :

He [Titchener] illustrated his argument with the example of a person who hastily glances across a street in preparation for crossing and is then momentarily distracted by the contents of a store window. On crossing the street, the person experiences false recognition as the feeling of having previously crossed that same street, a feeling of deja vu. By Titchener’s account, “the preliminary glance, which naturally connects with the crossing in a single, total experience, is disjoined from the crossing . . . and comes to consciousness separately as the memory of a previous passage”. This is described as the severing of “two phases of a single consciousness; the one is referred to the past; and the other, under the regular laws of memory, arouses the feeling of familiarity” (p. 425 from Titchener, 1928, A text-book of Psychology, Macmillan ).

Jacoby and Whitehouse attempted to produce such a “hastily glance” by briefly presented context words before each recognition test word. The paradigm worked as follows: Participants studied a list of words and then received a yes/no recognition test. Before each recognition test item, a masked context word (which on critical trials matched the test item) was presented long enough to consciously perceive the item (500 ms) or at a duration that was presumably unconscious (50 ms or 16 ms across Experiments 1 and 2). The important finding was that false alarms to lure items increased when the context words were presented at unconscious levels, compared with control words, but correct rejections to lure items increased when the context words were presented at longer durations. Jacoby and Whitehouse argued that these results are most consistent with a memory attribution process regarding the source of familiarity. When the primes are unconscious, the familiarity from the context words is attributed to oldness, whereas, when the participants are aware of the primes, they discount any familiarity produced by the primes, increasing their correct rejections. Jacoby and Whitehouse emphasized the importance of finding opposite patterns of results for the aware and unaware conditions, and as we shall see, *opposition of processes* is critical for Jacoby’s future work.

Jacoby, Woloshyn, and Kelley (1989) begin their paper with the following sentence:

“Folk wisdom suggests that we benefit from experience by consciously remembering those experiences and applying the knowledge gained from them to the current situation” (p. 115).”

In this paper, they demonstrate the power of unconscious influences on memory and the importance of attribution processes in a study that specifically runs counter to such “folk wisdom. They do not rely on recognition memory performance in this study but extend their work to judgments of fame for specific names, i.e., does the name  refer to a mildly famous person (e.g., Minnie Pearl) or is it a nonfamous name (e.g., Sebastian Weisdorf). In their second experiment, participants read aloud a series of 40 nonfamous names, either under divided attention (monitored auditory sequence of digits for target digit) or a full attention (without the secondary task). After reading the list of names, participants were specifically told that the names they just read were all nonfamous names. They then received a fame judgment task which included famous names, new nonfamous names that were not presented in the first phase of the experiment, or old nonfamous names that were presented in the first phase of the experiment. The critical finding was that, compared with new nonfamous names which were not presented earlier, participants were *more likely* to incorrectly call a nonfamous name famous under the divided attention conditions, whereas they were less likely to call a nonfamous name famous under the full attention condition. Jacoby, Woloshyn and Kelley argued that the results are consistent with an unconscious influence on memory in the divided attention condition. Specifically, participants could not attribute the increased familiarity from the earlier presentation under the divided attention conditions, and hence they decided the nonfamous names (e.g., Sebastian Weisdorf) were indeed famous. In contrast, for the full attention condition, because subjects were told that all of the previous names were nonfamous names, participants could discount any familiarity of the names due to their earlier presentation and use recollection of earlier presentation to know that the name is nonfamous. This study again nicely supports the two distinct processes used to drive memory performance (here, fame judgments) and produced another powerful example of showing *opposite* effects of conditions that rely on different aspects of memory, which leads us to the important next stage.

The papers published in 1989 were not only critical in showing dissociations of automatic and attentional mechanisms in memory performance, but they were also critical in emphasizing distinct memory “attribution processes” that depend on task demands. Specifically, different memory processes are used to attribute different sources to drive performance across different tasks. The emphasis on task demands and memory attributions from the participant’s perspective have also been influential in other domains in the literature. For example, the importance of task specific memory attributions has been extended to the literature on implicit vs. explicit memory tasks and attribution errors (misattributions) that underlie some false memories (see Jacoby, Kelley & Dywan, 1989, for detailed discussion of the importance of memory attributions).

## Process dissociation procedure (PDP; Jacoby, 1991)

The papers reviewed above capture a number of themes that led to Jacoby’s most highly cited paper, which afforded an attempt to *quantify* the contributions of attention-driven recollection and automatic familiarity based processes. Jacoby was very sensitive to ongoing debates in the literature regarding unconscious processing. This work was particularly relevant to Jacoby because he used the term “unconscious” frequently and, as noted, in both of the titles of the 1989 papers discussed above. The search for truly unconscious processing within experimental paradigms has been a long-standing and controversial topic since the work by McGinnies (1949) on perceptual defense, which was forcefully criticized along with other work on the topic by Adams (1957). Experimental psychologists were cautious about using the terms subliminal/unconscious due to methodological reasons that plagued much of this work. During the 1980s, there was a new wave of studies investigating semantic priming which purportedly provided evidence that one can indeed produce a semantic priming effect for targets even when the primes were below a consciousness threshold (e.g., Balota, 1983; Fowler et al., 1981; Marcel, 1983). However, Holender (1986) criticized these studies pointing out that one can question whether the primes were indeed below such a threshold (see also Holender & Duscherer, 2004). The search for truly subliminal/unconscious processing is central to discussions in both cognitive neuroscience (e.g., Dehaene, 2014), and philosophy (e.g., Fodor, 1985). If one finds evidence for truly unconscious processing, then this clearly would not be open to folk psychological explanations that would seem to hinge on conscious introspective processing.

Jacoby recognized this problem and realized that this is another example of lack of task purity. The tasks used for demonstrating unconscious processing are likely not process pure. This was a concern for Jacoby’s own work. That is, how can one be assured that the prime stimuli were not consciously processed in the brief presentation of primes in the Jacoby and Witherspoon paper and that the use of an auditory monitoring task eliminated awareness in the Jacoby, Woloshyn and Kelley papers, reviewed above. Given that a fundamental aspect of two process theories is the notion that there is a qualitative distinction between aware (conscious) and unaware (unconscious) processing, this critique was particularly troublesome. Specifically, is it not possible that some of the influences of “unaware” trials where actually “aware” trials that were not eliminated by the procedures? This is where Jacoby and colleagues emphasized the importance of finding opposing effects of the two processes. They argued that if the procedure for defining awareness was not airtight as Holender and others have suggested then how could such an explanation handle the evidence of opposing influences of the aware and unaware process on performance. Hence, Jacoby argued that a critical step to minimize the Holender critique was to identify conditions which a priori should show the two processes act *in concert* vs. conditions in which the two processes act *in opposition*. Jacoby again emphasized the lack of task purity and suggested that a useful approach would be to attempt to measure the *relative* contributions of unconscious automatic and conscious attention-based processes within a given task instead of across different tasks.

Jacoby’s insight was that if one could develop conditions in which the two processes act in concert and conditions in which the two processes act in opposition, one could actually estimate the relative contributions of each process to a given task performance. An important assumption here is that the two processes provide *independent* contributions to performance (see also Mandler, 1980). Consider a recognition task. As noted above, recognition performance presumably reflects a contribution of attention demanding recollection (R) of the previous episode and/or an automatic (unconscious?) boost in relative familiarity (F) due to its earlier presentation. In this sense, old/new recognition could be considered an inclusion (I) condition in which both processes act in concert. If the two processes are independent, then one could estimate the contributions of R and F from the following equation: I = R + F − (R × F). By itself, this is unsolvable based on performance because there are two unknowns. This is where the condition in which the two processes act in opposition is critical, referred to as exclusion (E) in this example. That is one can only respond based on recollection in this condition (e.g., respond “old” only if the item was in a particular context during its earlier presentation). If the two processes are indeed independent and both R and F contribute to performance in the inclusion condition and only R contributes to performance in the exclusion condition, then one could estimate the R contribution by simply taking the difference between the I and the E conditions (i.e., R = I − E). Once one obtains an estimate of R then one can use this to solve the estimate for F, in the earlier equation.

As noted, an important assumption of the PDP is that the two processes are independent. Jacoby argued that if this is the case, then one can test this by demonstrating invariance of both processes across multiple conditions. If invariance is held, then one should be able to show that under some conditions, the F estimate is invariant across multiple levels of R and under other conditions, the R estimate is invariant across multiple levels of the F process. He likened this to the highly influential signal detection theory (Green & Swets, 1966) in showing that discriminability can be demonstrated to hold constant across varying levels of bias, and bias can be demonstrated to hold constant across varying degrees of discriminability.

In the 1991 paper, Jacoby began providing empirical evidence using the PDP procedure by including both an inclusion condition (in which both F and R act in concert) and an exclusion condition (in which F and R act in opposition). Consider, for example, his third experiment. During the first phase of the experiment, participants either read or solved anagrams for a series of words. During the second phase of the experiment, participants heard and then pronounced each word aloud. The Inclusion test phase of the experiment required participants to respond “old” to any word that was previously presented in the experiment (i.e., read, heard, or solved as an anagram). In contrast, in the exclusion phase of the experiment, participants were instructed to only respond “old” for items that were heard during the second phase of the experiment and respond new to all other words. Here one can see that the exclusion condition places familiarity (due to an items earlier presentation in any condition) against the R process (due to recollection of items that were only heard earlier). With the results from this experiment in hand, Jacoby used the equations described above to obtain estimates of familiarity and recollection within the same recognition task in Experiment 3. In addition, he was able to compare these estimates to estimates from Experiments 1 and 2, which focused on the influence of divided attention on inclusion and exclusion conditions, respectively. Across the three experiments, Jacoby demonstrated that the estimates of recollection (reflected by the difference between Inclusion and Exclusion conditions) were highly dependent upon divided attention, whereas, the estimates of familiarity (based on the percentage of items in the exclusion condition that were not recollected), were relatively uninfluenced by divided attention (i.e., a demonstration of invariance of divided attention for familiarity estimates across varying levels of recollection). This was the first demonstration of how these estimates could be used in conjunction with a divided attention manipulation in an old/new recognition test and indicated that the estimates were relatively well-predicted by the demands of attention on the recollection estimate.

**Fig. 1:**
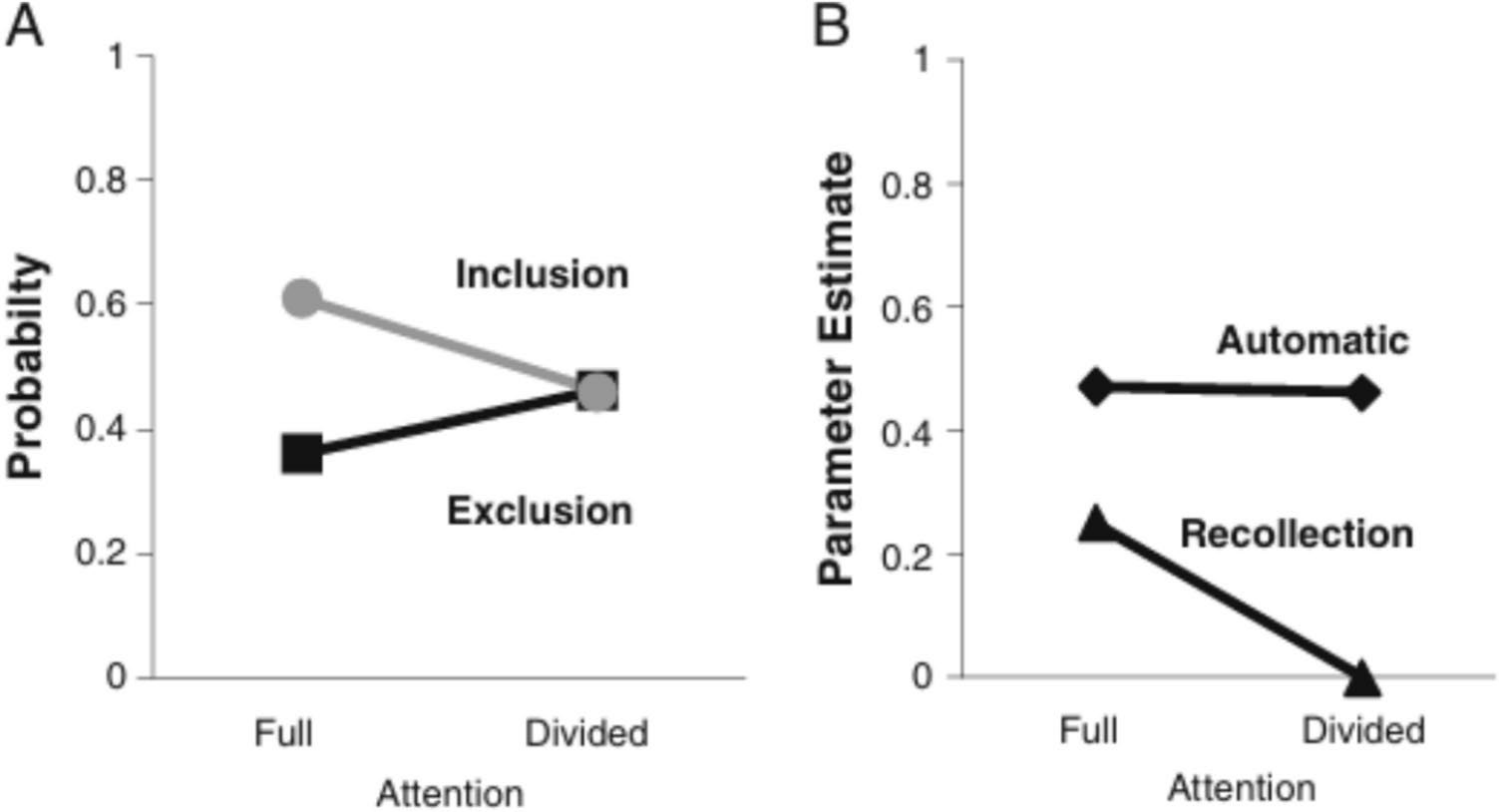
**A** Probability of completing a word stem with a studied word under inclusion and exclusion conditions, and (**B**) estimates of recollection and automatic influences of memory. Data from Exp. 1b of “Separating Conscious and Unconscious Influences of Memory: Measuring Recollection” by L. L. Jacoby, J. P. Toth, and A. P. Yonelinas, 1993, *Journal of Experimental Psychology: General, 122,* pp. 139–154. Copyright 1993 by the American Psychological Association

Below is a figure taken from Yonelinas and Jacoby (2012) capturing these relationships in a follow-up single experiment by Jacoby, Toth, and Yonelinas (1993). In the left panel one can see performance in the inclusion and exclusion conditions, similar to the Jacoby and Dallas (1981) paper. In the right panel one can see the estimates of recollection and familiarity based on the process dissociation procedure. As one can see in the right panel, only the recollection estimate is influenced by divided attention, whereas the automatic component is uninfluenced by this manipulation Fig 1.

The previous examples nicely demonstrate the power of Jacoby and colleagues’ use of invariance across tasks. In addition to the relatively isolated influence of divided attention on R, researchers have also demonstrated invariance by other procedures. For example, when one considers the speed of recognition decisions with response deadlines, there is evidence that fast responses appeared more likely to reflect F estimates whereas slower processes appeared more likely to reflect R estimates (e.g., Yonelinas & Jacoby, 1994, 1995). In addition, multiple studies of young vs. older adults have shown that aging influences R estimates and not F estimates (e.g., Hudson, 2008; Jennings & Jacoby, 1993). Remarkably, in a review of the literature available at that time, Jacoby et al. (1997) found that across 20 studies, the above experimental comparisons consistently yielded an influence on estimates of R (on average .24) with virtually no influence on estimates of F (.002). Although it does appear to be the case that there is more evidence of invariance of F across multiple levels of R, there are also examples of an effect of a variable on F but not on R (see, e.g., Jacoby et al., 1993).

### Challenges for PDP

Given its widespread use and relatively straightforward application, it is not surprising that the PDP approach garnered considerable attention along with some criticism. Probably, the most problematic is the rather strong assumption that the automatic and attention processes produce independent influences in a task (see Yonelinas & Jacoby, 2012, for a detailed discussion of this and other concerns). For example, Curran and Hintzman (1995) found consistent correlations between estimates of R and F in their experiments, which questions whether the two processes are independent. To counter this argument, Jacoby and Shrout (1997) showed that such correlations only occur when one collapses across items for a given participant or across participants for a given item. Jacoby and Shrout argued that the predictions from the PDP involve specific trials for specific items, and so when collapsing across items or participants correlations can be produced and indeed might be expected, which are beyond the scope of the PDP (see Hintzman & Curran, 1997, for a reply). In support of this argument, Rouder et al. (2008) used hierarchical analyses to come to the same conclusion regarding the problem with interpreting correlations when collapsing across items or participants. In another challenge to the PDP, Curran and Hintzman (1997) reported results that were inconsistent with predictions from the PDP. Specifically, increasing study time produced an increase in Recollection, but a surprising decrease in Familiarity. Jacoby (1998) argued that the instructional sets could have produced a generate-recognize strategy in the Curran and Hintzman (i.e., complete the first word that comes to mind followed by a recognition check), and indeed replicated the Curran and Hintzman results with their original instructions. However, when Jacoby used an instructional set that minimized the recognition check the results were consistent with the independence assumption. In addition, a related important assumption of the PDP approach is that the F process is the same in inclusion and exclusion conditions, and likewise the R process is also the same in inclusion and exclusion conditions. This is another strong assumption. One could conceive of crosstalk between the two processes or a third decision process that could take into consideration how the processes evolve over time. As Yonelinas and Jacoby (2012) have argued, some of the best evidence against this assumption is the extension to other experimental paradigms (beyond the original inclusion/exclusion paradigms, see below) and consistent utility of the PDP in explaining the results.

Clearly, the strong assumptions of the PDP have been quite controversial. This does not diminish the importance of the PDP in generating novel perspectives on different paradigms in the experimental literature. In this light, Jacoby emphasized that the use of converging operations (see Garner et al., 1956) across experimental paradigms are critical in providing confidence or lack thereof for a given theoretical account. Although one may never be able to fully “prove” the veracity of a theory (see Anderson, 1978), consistent patterns of results when experimental manipulations discriminate between different theories can increase one’s confidence in the utility of a theoretical perspective. In this light, the work by Jacoby and colleagues has been particularly powerful. For example, the distinction between familiarity and recollection processes has been used to account for changes in false recall across young, older adults, and individuals with early stage Alzheimer’s disease (e.g., Balota et al., 1999), and Jacoby’s recollection mechanism has been specifically used in Brainerd and Reyna’s (2002) recollection-rejection process in fuzzy trace theory. Although there are many other examples (see Yonelinas & Jacoby, 2012, for a review), I will focus on a brief review of how the use of converging operations has been critical in understanding performance in two well-developed domains of cognitive research, attentional control and the phenomenology of memory.

## PDP and Stroop performance

Stroop (1935) published one of the most influential papers in experimental psychology, providing a relatively simple experimental paradigm to investigate attentional control. In a typical Stroop task, participants are asked to name the color of a word which is either congruent (e.g., the word red in RED) or incongruent with the word (e.g., the word blue in RED). There are also often neutral conditions such as a non-color word (e.g., the word car in RED) or a sequence of non-alphabetic stimuli (e.g., &&&& in RED). The results from hundreds of studies have shown that color naming is faster in the congruent condition than the incongruent condition with the neutral condition often falling between the two conditions. One of the common accounts of the Stroop task is that word reading is faster than color naming and so the RT cost when the colors and words mismatch reflects interference from the incongruent word and the RT benefit reflects facilitation from the matching word. Although the congruent condition is faster than the incongruent condition, the degree of facilitation and interference compared with the neutral condition is inconsistent across studies with varying neutral conditions (e.g., noncolor words [car] or strings of characters [&&&]), however, facilitation effects are typically smaller than interference effects. Lindsay and Jacoby addressed the varying interference and facilitation effects across studies compared with the neutral condition. Given that there are differences across neutral conditions (see Jonides & Mack, 1984), this may not be surprising. For the present purposes, I will focus on Lindsay and Jacoby’s strategy to obtain PDP estimates from the congruent and incongruent conditions.

**Fig. 2:**
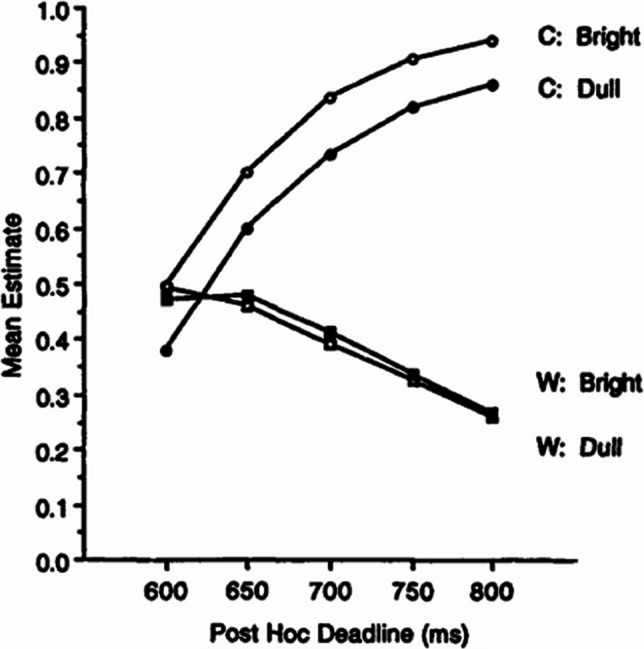
Process estimates from post hoc response latency deadlines taken from Lindsay and Jacoby’s (1994) manipulation of color purity. The estimates indicate that color purity only influences the color dimension with no influence on the controlled word dimension

Although the Stroop task was primarily developed to study attentional control, Lindsay and Jacoby (1994) argued that this paradigm is also ideally suited to apply the PDP procedure. As noted, a critical requisite of the PDP is to have both an inclusion condition in which processes act in concert and an exclusion condition in which processes act in opposition. Inclusion and exclusion conditions naturally fall from the Stroop task. Specifically, the congruent condition (word and color processes are the same) is an example of an inclusion condition and the incongruent condition is an example of an exclusion condition (word and color are in opposition). Hence, one might be able to apply the PDP equations to the Stroop conditions to obtain estimates of the automatic word reading process and the controlled color naming process. However, a problem with this approach is that response latency is typically the major dependent variable in Stroop and so one does not have accuracy measures typically used in PDP. In order to circumvent this problem, Lindsay and Jacoby used a deadline procedure that allows one to obtain accuracy estimates when participants are forced to respond at a short delay, along with an ad hoc partitioning of RTs into reaction time bins. The results from a series of experiments provided a dissociation between the contributions of a more automatic word dimension and a more controlled color dimension. For example, when the color of the stimulus was manipulated by presenting clear colored words vs. degraded colored stimulus, there is a large effect of the contributions of the automatic color dimension, but not the controlled word dimension (see Fig. 2).

In the third and fourth experiments, Lindsay and Jacoby manipulated the probability of congruent and incongruent trials. The common finding in the literature is that the Stroop effect is reduced in mostly incongruent conditions, compared with the mostly congruent trials. Lindsay and Jacoby predicted that in mostly incongruent trials, participants may be able to minimize the influence of the word dimension, via a controlled process. This is precisely the pattern obtained (i.e., there was no influence of mostly incongruent trials on the color dimension, but a strong influence on the word dimension; see Table 1). Thus, across experiments, they found that a manipulation of color clarity only influenced the color estimates, while a manipulation of proportion incongruent only influenced the word estimates.

**Table 1:** Color and word reading process estimates taken from Lindsay and Jacoby (1994)

	Process			
	Color naming		Word reading	
Measure	*M*	*SD*	*M*	*SD*
Experiment 3				
Most congruement	.84	0.22	.56	0.22
Most incongruement	.88	0.17	.30	0.16
Experiment 4 (count dots)				
Most congruement	.59	0.20	.53	0.18
Most incongruement	.58	0.25	.27	0.15

*Note.* Stroop proportion congruent manipulations only influenced the word dimension with no influence on the color dimension

The Lindsay and Jacoby study is important because it demonstrated the generality of the PDP to a different domain, attention, and showed how one can use a response deadline procedure and ad hoc RT binning to obtain measures of accuracy used to generate PDP estimates. As Lindsay and Jacoby acknowledge, the results from their study are consistent with models that color and word reading processes involve parallel processes (e.g., Cohen et al., 1990; Logan, 1980), and was not intended to discriminate amongst the computational models, but rather provided a procedure to estimate the relative contributions of automatic and attentional processes to performance. This extension has been quite useful. For example, based on the Lindsay and Jacoby approach, Spieler et al. (1996) used response latency binning to obtain estimates for word and color estimates, and found that the effect of aging was localized to the contributions of the attention demanding word estimate, while the effect of Alzheimer’s disease reflected impairments in both the automatic color and attention demanding word estimates.

### Remember/Know reports

In his classic paper, Tulving (1985) tackled an important aspect of memory (i.e., the conscious state of a memory event). For example, one may respond correctly to an “old” item on a recognition test, but this could be due to an experience of familiarity and/or an experience of recollection. The trick is how to study these phenomenological states. Tulving decided to try and capture these two different conscious states by developing the Remember/Know procedure. The procedure is to have participants make old/new recognition decisions and afterwards report on their phenomenological state for “old”. Specifically, in the simplest paradigm, participants are instructed that after they make an “old” response to an item, they are to indicate if their response is based on a specific detail of the item’s earlier presentation (Remember) or are calling an item old because the item seems familiar (Know). Interestingly, the R/K distinction has shown dissociations that appear to be consistent with two process theory. For example, Gardiner (1988) found that depth of processing manipulations influenced remember judgments but did not influence know judgments. This led him to conclude that R judgments reflect more recollective processes whereas K judgments reflect more perceptual processes.

Because the R/K distinction appears to map onto recollection and familiarity, one might expect some convergence across the estimates between PDP estimates and R/K reports. However, as Yonelinas and Jacoby (1995) pointed out, there is a major difference between the two approaches. Specifically, R/K reports rely on an exclusivity assumption—that is, participants can only respond R (recollection) or K (familiarity). This runs counter to the major assumption in the PDP, since any task/response may reflect both attention based (recollection) and automatic (familiarity) processes. In order to address this issue, Yonelinas and Jacoby reported three experiments that involved a list discrimination procedure and a size congruency manipulation. The major finding was that when one used PDP estimates in Experiment 1, size congruency influenced both the recollection estimate and the familiarity estimate. However, when one used the R/K estimates, one found that recollection (as reflected by R judgments) increased with size congruency but surprisingly, familiarity (as reflected by K judgments) actually decreased. This replicated an earlier finding by Rajaram and Coslett (1992). Thus, there was a clear difference between the PDP estimates and the R/K estimates.

In order to reconcile this discrepancy, Yonelinas and Jacoby developed the IRK procedure. They argued that one should avoid the exclusivity assumption of simple R/K responses and use the independence logic of the PDP to obtain estimates. The basic argument is that one must correct for the percentage of items available for a K response—that is, 1 – R. Hence, F = K/(1 − R). When this correction was made for the R/K estimates, the results from both Experiments 1 and 2 converged on the notion that size congruency facilitated both R and K judgments, the same pattern obtained by the PDP estimates. This convergence across objective estimates and subjective estimates is quite powerful and has been important in increasing confidence in using  Jacoby's PDP framework.  As Yonelinas and Jacoby (1995) also pointed out this convergence across objective and subjective measures is also problematic for simple strength models in suggesting that R responses are merely stronger memory traces than K responses (see, however, Wixted & Mickes, 2010, for a continuous dual-process model of R/K judgments).

## PDP+ and the Jacoby lineage

When considering the influence of Jacoby on understanding memory, attention and consciousness, it is also important to consider the influence of Jacoby on students, postdocs, and colleagues. Here, I will focus briefly on the important work of Larry’s graduate student and colleague, Andy Yonelinas, because this naturally flows from the PDP perspective. I will briefly discuss here two domains in which Yonelinas led to important developments, ROC analyses and Cognitive Neuroscience.

### ROC analyses

**Fig. 3:**
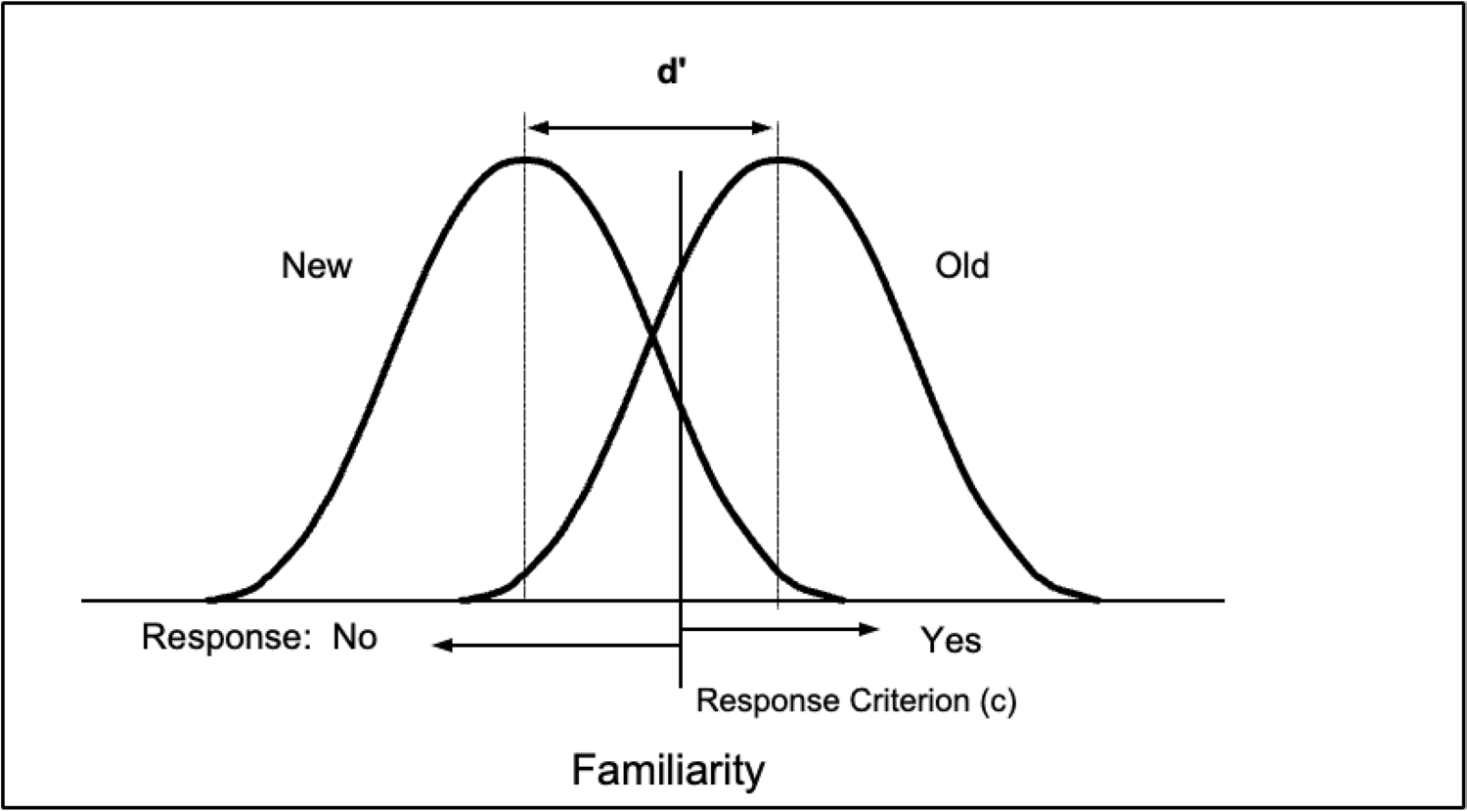
Distributions of old and new stimuli along a familiarity dimension; *d′* reflects the discriminability between the mean of the old distribution and the mean of the new distribution in *z*-scores. Response criterion, *c*, reflects the threshold along the familiarity dimension for making an “old” or “new” response, taken from Yonelinas (2001)

**Fig. 4:**
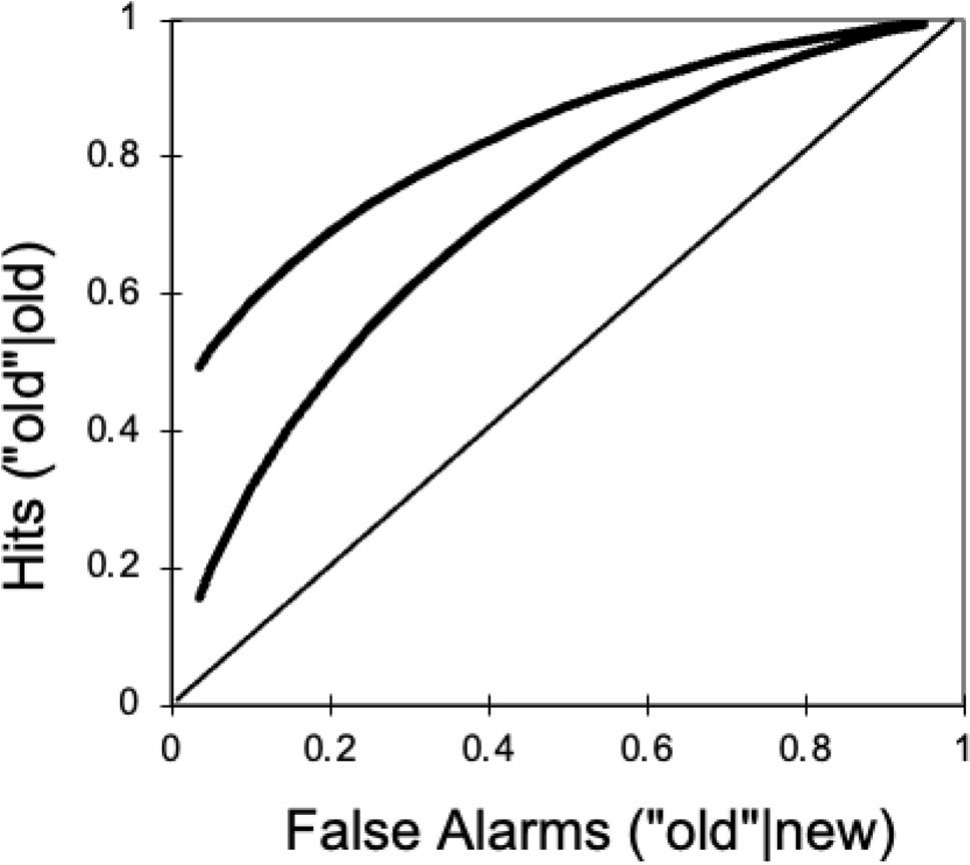
Receiver operating characteristics based on Fig. 3, which plots the false-alarm rate against the hit rate. The prediction from a global strength model might be a symmetrical ROC, middle line, but one often finds an asymmetrical ROC, as reflected by the top function, adapted from Yonelinas (2001)

Signal Detection Theory (SDT; Green & Swets, 1966) is an analytic tool that has been used across a wide range of decision situations, wherein one can estimate both sensitivity and bias in decisions. ROC analysis naturally falls from Signal Detection Theory and expresses the relationship between hit rates for old items and false alarm rates for new items across varying levels of confidence. For example, Fig. 3 displays Gaussian distributions reflecting old and new items along a familiarity dimension in a standard SDT model. If this model were correct, and one plots Hits vs. Fals alarms across different levels of confidence, then one would expect a symmetrical ROC, as displayed in middle curved function of Fig. 4. Symmetry in the ROCs would naturally fall from global memory strength models such as MINERVA (Hintzman, 1986). However, it is most often the case that ROCs are not symmetrical but are asymmetrical, as displayed in the top function in Fig. 4 (e.g., see Ratcliff et al., 1992, for a discussion). Yonelinas argued that this asymmetry naturally falls from a two-process model involving familiarity and recollection. Specifically, the asymmetry reflects the contribution of recollection which supports high confidence targets, with very few false alarms. Hence, Yonelinas developed the dual-process SDT model, which assumes that the familiarity process is reflected by *d′*, as in the basic SDT model, with an additional parameter reflecting the probability an item is recollected, thereby producing the asymmetry (see Yonelinas & Parks, 2007, for a review).

**Fig. 5:**
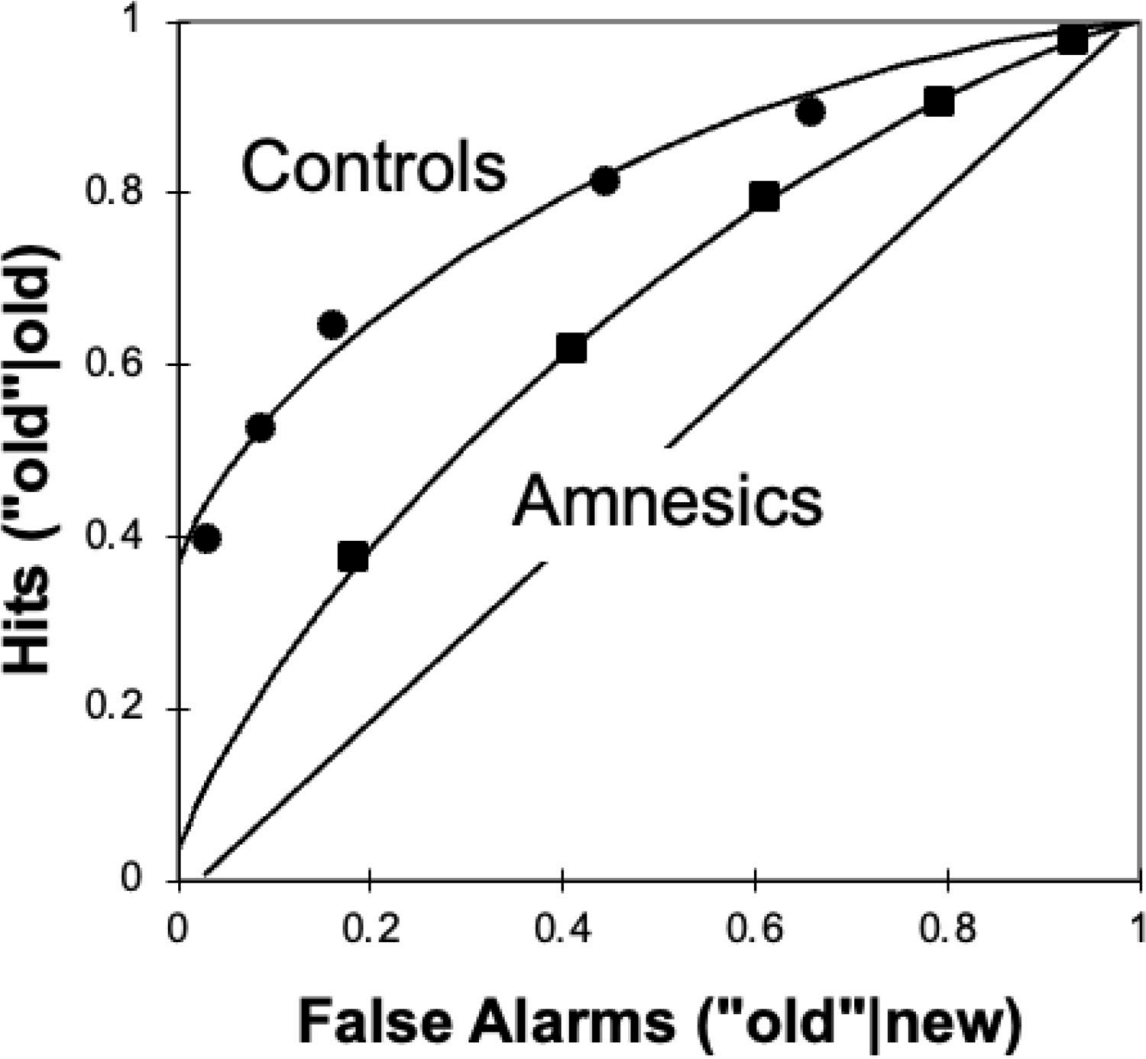
ROCs of amnesics and Controls taken from Yonelinas et al. (1998). Note that the Controls are more asymmetric than the amnesics

**Fig. 6:**
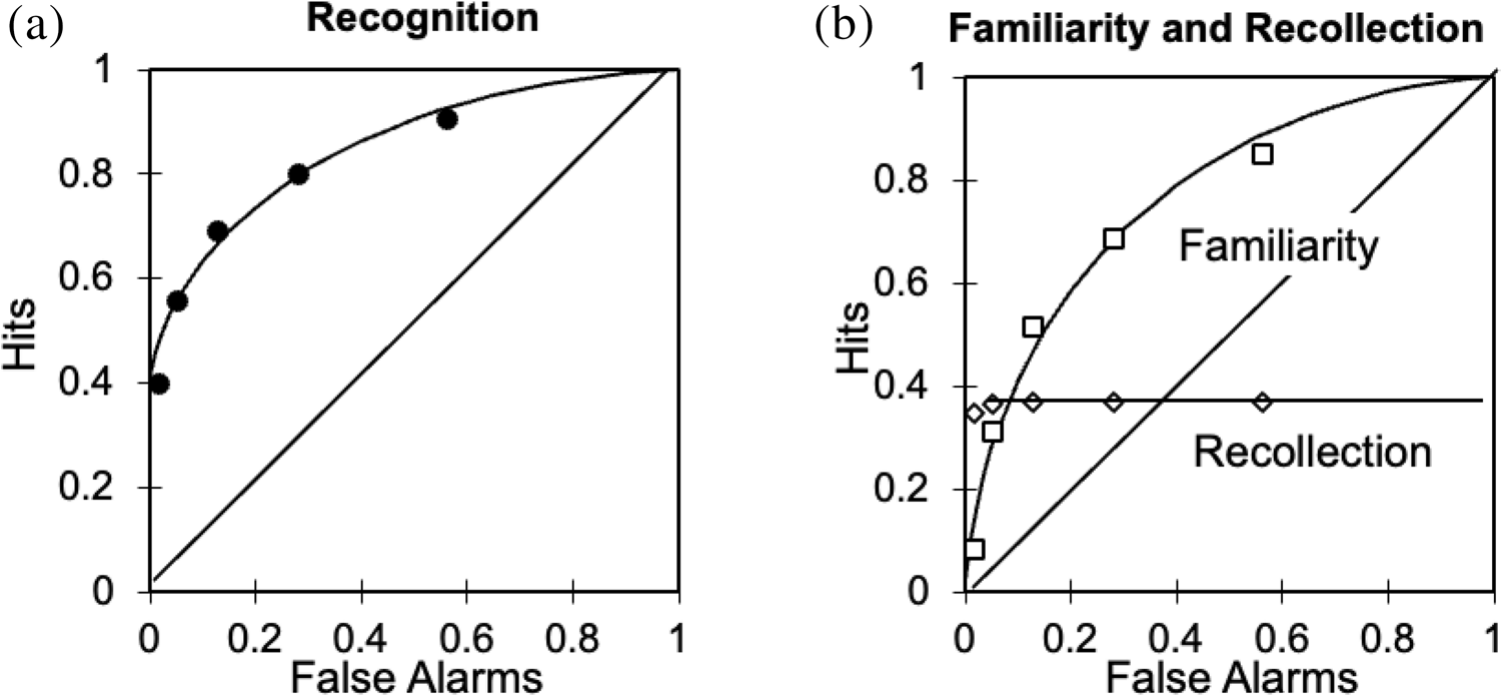
(**a**) Recognition ROCs taken from Yonelinas (2001). (b) are the ROCs based on PDP estimates from Remember/Know judgments on the same data. As shown, the familiarity estimates appear symmetrical, whereas, the recollection estimates are flat, showing a dissociation at the 0 false alarm rate

The dual process SDT model has received support from a number of empirical demonstrations. First, in the original Jacoby (1991) study described above, there were recognition memory results along with confidence ratings available. Yonelinas (1994) plotted the ROCs across participants along with the standard PDP estimates. The results indicated that as recollection estimates from PDP increased the estimates of the degree of asymmetry also increased, whereas, when both familiarity and recollection increased, there was no increase in the estimates of asymmetry because the two processes presumably offset each other in the ROCs. Second, one might argue that amnesic individuals may have a familiarity signal, but little recollection available (see, e.g., Huppert & Piercy, 1976). If this is the case, then one would predict that the ROCs for amnesics would be curvilinear and symmetrical. As shown in Fig. 5, Yonelinas et al. found precisely this pattern. Finally, when one considers the R/K phenomenological reports described above, one might expect that R items (reflecting recollection) would be more asymmetrical than for K items, reflecting familiarity. Indeed, as shown in Fig. 6, this is what Yonelinas (2001) observed.

There are clearly alternative views of the asymmetric ROCs. Wixted (2007a, 2007b) and others have noted that the asymmetry in ROCs naturally falls from the fact that there is greater variance for the Old distribution compared with the New distribution, simply because the variance due to “oldness” is added to the variance for the new distribution along a memory strength dimension. Once one takes into consideration this difference in variance, then one can use a single *d′* estimate to capture recognition performance. This would be supportive of single process models (e.g., MINERVA and TODAM) that reflect a single memory strength value to drive performance. Based on earlier work (e.g., Wixted & Stretch, 2004), Wixted (2007a, b) argued for a dual process signal detection model by acknowledging there are two types of processes involved in recognition (familiarity and a recollection process). The major difference in this perspective is that both familiarity and recollection are combined at the time of decision, as opposed to assuming that either familiarity or recollection is used. It is clearly beyond the scope of this paper to adjudicate between the two perspectives (see Pratte & Rouder, 2011, 2012, for a discussion); however, there is little doubt that the dual process SDT model of Yonelinas has generated considerable research in understanding the potential causes of ROC asymmetry.

### Neural substrates of recollection and familiarity

Although Jacoby recognized the importance of comparing different participant groups (amnesics vs. controls, young vs. old, healthy old vs. Alzheimer’s disease patients), and had a very active research program in aging, his interest in the specific neural substrates of recollection and familiarity was more from the sidelines than an active participant. In contrast, Yonelinas and colleagues have developed a rich research program specifically aimed at isolating the neural substrates of recollection and familiarity. Given that there are consistent behavioral differences between recollection and familiarity, it may not be surprising that there are distinct neural signatures. However, isolating the specific contributions has not been a simple enterprise. Since amnesic individuals with medial temporal lobe (MTL) damage often have a selective deficit in direct recollective tests of memory but relatively preserved performance on indirect tests, this was taken as evidence that the MTL reflected recollective processing and areas outside of the MTL reflected familiarity based processing. However, it soon became apparent that MTL damage can produce deficits in both recollection and familiarity processing, albeit the latter to a smaller degree (see Yonelinas et al., 1998, for a review).

The MTL is a relatively large area with multiple subregions, and hence it is critical to estimate the function of these subregions, and damage to MTL may not be isolated to one subregion. Hence, there are often debates regarding the purity of lesions. One pattern that is quite consistent across studies is that isolated effects in one subregion, the hippocampus, does appear to produce relatively isolated effects on recollection (Aggleton et al., 2005; Yonelinas et al., 2004). Remarkably, Bowles et al. (2007) also studied a patient who had relatively isolated damage to the perirhinal cortex and found that familiarity was disrupted but not recollection (see also Wolk et al., 2011). Of course, human lesion data can often be complicated by the degree to which there are isolated neural subregions involved. In this light, it is noteworthy that there was converging  evidence from animal studies which can better control lesion location suggesting that the hippocampus produces deficits in recollection while leaving familiarity relatively intact (see Eichenbaum et al., 2007; Fortin et al., 2004, for a review).

Although the perspective that the hippocampus only supports recollection has received support from converging studies, there is also controversy in this area. For example, Wais et al. (2006) have provided evidence that both familiarity and recollection can be subserved by the hippocampus. Also, Squire et al. (2007) acknowledge that both recollection and familiarity signals are important for recognition (consistent with the dual process signal detection model cited above), but argue from both human and animal studies that both the perirhinal cortex and the hippocampus can subserve both signals. In contrast, Eichenbaum et al. (2007) provided a review continuing to support the distinction between the roles of these areas for recollection and familiarity. Of course, this debate does not preclude the role of other areas that may play a role in recollection and familiarity, and indeed there appears to be evidence of widespread distinct networks involved in recollection and familiarity, including frontal areas (see Yonelinas et al., 2005). Whatever the final answers are to the functions of specific neural areas, Jacoby’s emphasis on understanding the role of attention, consciousness, and memory attribution processes, along with careful task analyses, provides guidance in interpreting the functions of these complex networks.

### Summary

The present contribution focuses on the historical context and evolution of Jacoby’s dual process theory, along with a brief discussion of extensions from the work by Yonelinas. Jacoby relied on principles of “the myth of task purity,” task invariance, converging operations, and careful experimental designs to tackle the contributions of an automatic familiarity based processes and an attention demanding recollective process to better understand the relationship among attention, memory, and consciousness. This article focuses primarily on a very active 25 year period in the history of experimental psychology. The goal is to capture the energy and zeitgeist that led to the evolution of the PDP, and its remarkable utility in better understanding the relationship between attention and memory. Of course, this is only one perspective by a colleague working during this same period, the breadth of his influence is reflected by the remaining contributions to this special issue.

## Data Availability

Not applicable.
